# Detection of micrometastasis by cytokeratin 20 RT-PCR is limited due to stable background transcription in granulocytes

**DOI:** 10.1038/sj.bjc.6690778

**Published:** 1999-11

**Authors:** R Jung, K Petersen, W Krüger, M Wolf, C Wagener, A Zander, M Neumaier

**Affiliations:** Departments of Clinical Chemistry, Bone Marrow Transplantation, Medical Clinic, University Hospital Eppendorf, Hamburg 20246, Germany

**Keywords:** RT-PCR, micrometastasis, cytokeratin 20, standardization, background transcription

## Abstract

The reverse transcription polymerase chain reaction (RT-PCR) amplification of cytokeratin 20 (CK20) mRNA is considered a promising candidate method for the detection of circulating tumour cells in bone marrow and peripheral blood of cancer patients. In this study we have investigated the diagnostic specificity of the CK20 mRNA detection in samples from healthy donors (HD; *n* = 33), intensive care units patients (ICU; *n* = 20) and bone marrow obtained from patients suffering from chronic inflammatory diseases (CID; *n* = 14). RNAs purified from stabilized lysates showed positive results in 24% of the HD group (8/33), 35% of the ICU group (8/20) and in 40% of the CID group (5/14). The use of Ficoll gradients to separate nucleated cells completely restored the specificity of this CK20 RT-PCR assay. The CK20-expressing cells are positively identified to belong to the granulocyte fraction of leucocytes, which appear to express the gene on a background level. Our results demonstrate for the first time that CK20 mRNA expression is not limited to epithelium. Its occurrence in normal granulocytes has to be considered in tests designed to detect circulating cancer cells or micrometastases. © 1999 Cancer Research Campaign


*British Journal of Cancer* (1999) **81**(5), 870–873
© 1999 Cancer Research Campaign
Article no. bjoc.1999.0778

### Detection of micrometastasis by cytokeratin 20 RT-PCR is limited due to stable background transcription in granulocytes

**R Jung[1], K Petersen[1], W Krüger[2], M Wolf[3], C Wagener[1], A Zander[2] and M Neumaier[1]**

Departments of [1]Clinical Chemistry, [2]Bone Marrow Transplantation, [3]Medical Clinic, University Hospital Eppendorf, 20246 Hamburg, Germany

**Summary** The reverse transcription polymerase chain reaction (RT-PCR) amplification of cytokeratin 20 (CK20) mRNA is considered a promising candidate method for the detection of circulating tumour cells in bone marrow and peripheral blood of cancer patients. In this study we have investigated the diagnostic specificity of the CK20 mRNA detection in samples from healthy donors (HD; $n = 33$), intensive care units patients (ICU; $n = 20$) and bone marrow obtained from patients suffering from chronic inflammatory diseases (CID; $n = 14$). RNAs purified from stabilized lysates showed positive results in 24% of the HD group (8/33), 35% of the ICU group (8/20) and in 40% of the CID group (5/14). The use of Ficoll gradients to separate nucleated cells completely restored the specificity of this CK20 RT-PCR assay. The CK20-expressing cells are positively identified to belong to the granulocyte fraction of leucocytes, which appear to express the gene on a background level. Our results demonstrate for the first time that CK20 mRNA expression is not limited to epithelium. Its occurrence in normal granulocytes has to be considered in tests designed to detect circulating cancer cells or micrometastases. © 1999 Cancer Research Campaign

**Keywords:** RT-PCR; micrometastasis; cytokeratin 20; standardization; background transcription

Presence of tumour cells shown by immunocytochemical detection in bone marrow has been convincingly correlated with a decline in disease-free interval and prognosis (Diel et al, 1996; Pantel et al, 1996). Specifically, antibodies against cytokeratins (CK), a family of cytoskeletal proteins expressed in epithelial cells, have been used successfully in various cancers. As an alternative, tumour cells may be detected at extremely low frequencies using high sensitivity reverse transcription polymerase chain reaction (RT-PCR) assays. While some haematological malignancies and non-epithelial tumours can be detected using RT-PCR of tumour-specific translocational gene fusions (Downing et al, 1993, 1995; Hiraga et al, 1998), the detection of epithelium-derived cancer cells is primarily based on the amplification of either tissue-specific mRNA expression, e.g. tyrosinase or prostate-specific antigen (PSA) (Gomella et al, 1997; Ghossein et al, 1998), or lineage-specific mRNAs like the cytokeratins that characterize cells of epithelial origin. Assays designed for the detection of epithelial cells mainly use mRNA of the cytokeratins 18, 19 and 20 (Kruger et al, 1996; Tschentscher et al, 1997; Futamura et al, 1998; Wyld et al, 1998).

The detection of micrometastasis by cytokeratin RT-PCR is difficult for a number of reasons: cytokeratin 18 (CK18) and cytokeratin 19 (CK19) are expressed in a variety of epithelia of different origins (Moll et al, 1982) and expression of their respective mRNAs has been reported in non-epithelial cells (Traweek et al, 1993). Furthermore, due to the existence of processed CK pseudogenes in the human genome, specific amplification may originate from residual nuclear DNA in sample preparations (Neumaier et al, 1995; Tschentscher et al, 1997). In contrast, the expression of CK20 is more restricted and no processed pseudogenes have been found (Moll et al, 1993).

In a number of clinical CK20 RT-PCR studies, comparable diagnostic specificities have been achieved for conventional (Burchill et al, 1995), nested RT-PCR (Soeth et al, 1996) and even double-nested RT-PCR (Funaki et al, 1998) systems. Recent studies have assigned clinical significance and prognostic value to the detection of CK20 mRNA-positive cells in bone marrow and peripheral blood samples of breast, gastric, pancreatic and colorectal cancer patients (Soeth et al, 1996, 1997; Bostick et al, 1998; Weitz et al, 1998). Initiated by our interest in influential factors in RT-PCR diagnostics (Jung et al, 1997*a*, 1998) and the observation of positive RT-PCR results from normal specimens, we have, in this study, systematically investigated the occurrence of illegitimate transcription of CK20 mRNA and its influence on the specificity of RT-PCR assays.

### MATERIALS AND METHODS

### Specimens

EDTA-blood samples were obtained from healthy volunteers (HD) and from intensive care unit (ICU) patients with no evidence of malignant disease. To avoid contamination with skin epithelial cells, second draws were used in the HD group and skin incisions were performed before marrow aspiration (CID group). In the ICU group, the samples were obtained from patients using an arterial catheter. The ICU-samples were processed using two different protocols. Of the whole blood specimens, 1 ml was immediately stabilized in 5 ml 6 M guanidiniumisothiocyanate (GIT), and 1 ml of the same sample was used for Ficoll plaque density gradient

*Received 25 January 1999*
*Revised 12 May 1999*
*Accepted 13 May 1999*

*Correspondence to:* M Neumaier

**Table 1**

| No. | Diagnosis | Serum CRP (mg $l^{-1}$) | Granulocytes (mg $\mu l^{-1}$) | Lysed whole blood | Lysed mononuclear cells |
|---|---|---|---|---|---|
| 1 | Cardiac arrest | 129 | 10.3 | pos./pos. | neg./neg. |
| 2 | Diabetes mellitus | 342 | 7.7 | pos./pos. | neg./neg. |
| 3 | Pancreatitis | 122 | 11.2 | neg./pos. | neg./neg. |
| 4 | Coronary artery disease | 125 | 6.4 | neg./neg. | neg./neg. |
| 5 | Pneumonia | 248 | 19.6 | neg./pos. | neg./neg. |
| 6 | Pneumonia | 168 | 13.6 | neg./neg. | neg./neg. |
| 7 | Pulmonary embolism | 300 | 10.9 | neg./neg. | neg./neg. |
| 8 | Septicaemia | 107 | 10.2 | neg./neg. | neg./neg. |
| 9 | Myocardial infarction | 22 | 19.4 | pos./pos. | neg./neg. |
| 10 | Septic inflammation | 28 | 4.3 | neg./neg. | neg./neg. |
| 11 | Pneumonia | 139 | 6.3 | neg./neg. | neg./neg. |
| 12 | Coronary artery disease | 61 | 7.1 | pos./pos. | neg./neg. |
| 13 | Peritonitis | 148 | 6.7 | neg./neg. | neg./neg. |
| 14 | Drug intoxication | 32 | 6.1 | neg./neg. | neg./neg. |
| 15 | Pancreatitis | 87 | 4.6 | neg./neg. | neg./neg. |
| 16 | Ulcerative colitis | 21 | 3.7 | neg./neg. | neg./neg. |
| 17 | Pneumonia | 82 | 6.5 | neg./neg. | neg./neg. |
| 18 | Aspiration pneumonia | 146 | 2.3 | neg./neg. | neg./neg. |
| 19 | Pneumonia, hepatitis | 158 | 11.9 | pos./pos. | neg./neg. |
| 20 | Intoxication, pneumonia | 10 | 10.1 | pos./pos. | neg./neg. |
| | CK20 mRNA positivity rate (%) | | | 40% | 0% |

neg, negative; pos, positive.

centrifugation according to the manufacturer's instructions (Amersham-Pharmacia, Freiburg, Germany). The upper layer containing the mononucleated cells was lysed in 6 M GIT-solution prior to RNA purification.

One sample (5 ml) of a healthy donor was processed in the same way as the patients' specimens described above. Additionally the layer containing the polymorphonuclear (granulocyte) fraction was further processed by osmotic erythrocyte lysis and subsequent wash steps to remove inhibitory haemoglobin from the cell fraction prior to CK20 RT-PCR amplification.

### Granulocytes count

Granulocytes were differentiated and counted using VCS-technology with a routine Coulter-analyser.

### RNA purification

Total RNA was purified from all specimens using the standard acid phenol–chloroform extraction method (Chomczynski and Sacchi, 1987). Nucleic acid concentrations were verified by spectrophotometry at 260 nm and 280 nm wavelengths, and approximately 2 μg total RNA were used for subsequent analysis as described previously (Gerhard et al, 1994). mRNA integrity was checked by β2-microglobulin RT-PCR as described previously (Jung et al, 1997*b*).

### RT-PCR assay

CK20 RT-PCR assays were performed as published (Soeth et al, 1996). Each sample was investigated twice and scored positive, if at least in one assay a specific amplification occurred. Positive samples were re-examined in RT-PCR-reactions without reverse transcriptase to exclude: (i) contamination with PCR-products or (ii) amplification of processed pseudogene sequences from contaminating nuclear DNA. The analytical sensitivity of the assay was assessed by limiting dilutions of HT 29 cells in $10^6$ Chinese hamster ovary cells (CHO). For the sensitivity limit in this study of ten HT29 cells in $10^6$ CHO-cells a 100% reproducibility was reached. In contrast to human blood leucocytes, CHO cells do not express CK20-like sequences to interfere with the HT29 signal and are therefore suitable for the dilution experiment.

### Statistical analysis

Statistical significance was calculated using the two-tailed unpaired *t*-test. Values are means with standard error.

### RESULTS AND DISCUSSION

A total of 67 specimens from healthy donors (HD; $n = 33$), patients suffering from inflammatory disease (CID; $n = 14$) and patients from an intensive care unit (ICU; $n = 20$) were investigated in this study using an assay designed to amplify CK20 mRNA. In the originally investigated stabilized lysates from bone marrow of CID patients ($n = 14$) and peripheral blood of HD ($n = 33$) we had encountered positive results in 35% and 24% respectively. This represents a markedly lower diagnostic specificity than has been previously reported for CK20 detection by RT-PCR (Burchill et al, 1995; Soeth et al, 1997; Funaki et al, 1998). To verify these results we investigated the interference of mRNA from nucleated blood cells on the CK20 RT-PCR specificity in a panel of peripheral blood samples from ICU patients with non-malignant conditions (Table 1). In addition to the blood lysate preparation, the specimens were subjected to a standard Ficoll-plaque density gradient separation of the mononuclear and granulocyte cell fraction. While 24% of the blood lysates from the HD group were found positive, the diagnostic unspecificity observed in the bone marrow lysates (35%) was very similar to that found in the peripheral blood lysates obtained from the ICU patient group (40%; Table 1).

© 1999 Cancer Research Campaign

**Table 2** Comparison of granulocyte counts and C-reactive protein (CRP) serum levels between CK20-positive and CK20-negative ICU-patients (values: mean ± s.e.)

| | CK20 RT-PCR-positive | CK20 RT-PCR-negative |
|---|---|---|
| Granulocytes $\mu l^{-1}$ | 12.3 ± 4.8 | 6.6 ± 3.1 |
| CRP mg $l^{-1}$ | 136 ± 113 | 115 ± 76 |

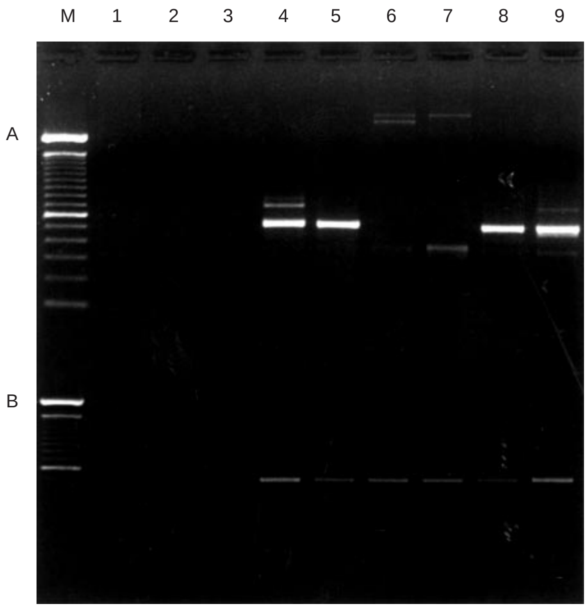

**Figure 1** Representative result of Cytokeratin 20 RT-PCR (**A**) of white blood cell subpopulations separated using Ficoll-paque density gradient centrifugation. Lane M: Molecular base pair marker (100 bp); lanes 1–3: negative controls; lanes 4 and 5: RNA purified from granulocytes; lanes 6 and 7: RNA purified from lymphocytes; lanes 8 and 9: positive controls. (**B**) β2-microglobulin RT-PCR, samples as listed above

Among the samples found positive by duplicate testing, 3 and 5 showed specific amplification products in one or both tests respectively. This variability is explained by stochastic effects at the limit of analytical sensitivity as has been discussed in detail by Jung et al (1997).

The positivity rate in the ICU patients whole blood lysates correlated with the number of granulocytes in the sample. Specifically, the mean granulocyte count per microlitre was significantly higher in the group of CK20-positive ICU-patients compared to the group of CK20-negative ICU-patients (12.3 ± 4.8 vs 6.8 ± 3.1 granulocytes $\mu l^{-1}$; $P < 0.02$; Table 2). The diagnostic specificity of the CK20 RT-PCR test was completely restored, when the specimen was subjected to Ficoll-plaque centrifugation prior to further analysis. Ficoll density gradient centrifugation separates two main white blood cell subpopulations: (i) lympho- and monocytes in the upper layer, and (ii) granulocytes above and in the red blood cell fraction. As shown in Figure-1, RT-PCR assays performed on the fraction of mononucleated cells were negative. In contrast, the CK20-expressing cells were positively identified in the granulocyte fraction of blood leucocytes previously depleted of erythrocytes by osmotic lysis.

The frequency of positive CK20 results was found to correlate significantly with the granulocyte count ($P < 0.02$). This is consistent with the observation that HD showed 24% positive samples, while 40% positives were found in the ICU patient group. This background mRNA expression in non-cancer patients and healthy individuals appears to be different from the illegitimate transcription occasionally found for other mRNA targets. We have recently shown in vitro that leucocytes can be induced by cytokines and growth factors to express mRNA targets used for the detection of circulating cancer cells. For example, the mRNA expression of the human carcinoembryonic antigen (CEA) is specifically inducible by interferon-γ, but not by interleukin (IL)-3, IL-6, granulocyte colony-stimulating factor, granulocyte–macrophage colony-stimulating factor or SCF. On the other hand, an induction of CK19 mRNA expression can be observed after 7 days of tissue culture without the addition of any of these cytokines (Jung et al, 1998). In contrast, none of the cytokines used was able to induce CK20 mRNA expression in this system (data not shown). Together with the correlation to the granulocyte count in the sample, this suggests a low abundance constitutive-type of CK20 mRNA expression in these cells in the peripheral blood. This interpretation is further corroborated by the observation that the positivity in specimens obtained from non-malignant patients does not correlate with inflammatory reactions or acute phase markers as judged by serum concentrations of the C-reactive protein (Table 2).

Our results demonstrate for the first time that CK20 mRNA expression is not limited to epithelial cells, but also occur in normal granulocytes. The data also suggest that CK20 mRNA is stably expressed at low levels in these cells rather than modulated under conditions of clinical illness. Studies using quantitative CK20 RT-PCR have to be performed to answer this question conclusively. However, these results allow the delineation of rational CK20 RT-PCR assay designs with respect to the clinical materials used, i.e. peripheral blood, bone marrow or other bodily fluids. Firstly, assays based on enriched mononucleated cells as sample material require no knowledge of the granulocyte count. On the other hand, disadvantages of the frequently-used Ficoll density gradient separation include the potential loss of malignant cells and difficulty to standardize the procedure. Secondly, for assays using lysates of whole blood or bone marrow as a mRNA, the number of granulocytes has to be determined. For those assays, cut-off values can be readily defined based on leucocyte counts. In contrast to mRNA targets that are subject to transcriptional modulation under different clinical conditions, the use of CK20 mRNA may provide a significant diagnostic advantage. Studies are now underway to establish, in a quantitative format, the cut-off values to improve the diagnostic specificity in the detection of minimal residual disease in CK20-positive human cancers.

### ACKNOWLEDGEMENT

This work is part of the MD doctoral thesis of KP.

### REFERENCES

Bostick PJ, Chatterjee S, Chi DD, Huynh KT, Giuliano AE, Cote R and Hoon DS (1998) Limitations of specific reverse-transcriptase polymerase chain reaction markers in the detection of metastases in the lymph nodes and blood of breast cancer patients. *J Clin Oncol* **16**: 2632–2640

Burchill SA, Bradbury MF, Pittman K, Southgate J, Smith B and Selby P (1995) Detection of epithelial cancer cells in peripheral blood by reverse transcriptase-polymerase chain reaction. *Br J Cancer* **71**: 278–281

 *© 1999 Cancer Research Campaign*

Chomczynski P and Sacchi N (1987) Single-step method of RNA isolation by acid guanidinium thiocyanate-phenol-chloroform extraction. *Anal Biochem* **162**: 156–159

Diel IJ, Kaufmann M, Costa SD, Holle R, von Minckwitz G, Solomayer EF, Kaul S and Bastert G (1996) Micrometastatic breast cancer cells in bone marrow at primary surgery: prognostic value in comparison with nodal status. *J Natl Cancer Inst* **88**: 1652–1658

Downing JR, Head DR, Parham DM, Douglass EC, Hulshof MG, Link MP, Motroni TA, Grier HE, Curcio-Brint AM and Shapiro DN (1993) Detection of the (11;22)(q24;q12) translocation of Ewing's sarcoma and peripheral neuroectodermal tumor by reverse transcription polymerase chain reaction. *Am J Pathol* **143**: 1294–1300

Downing JR, Khandekar A, Shurtleff SA, Head DR, Parham DM, Webber BL, Pappo AS, Hulshof MG, Conn WP and Shapiro DN (1995) Multiplex RT-PCR assay for the differential diagnosis of alveolar rhabdomyosarcoma and Ewing's sarcoma. *Am J Pathol* **146**: 626–634

Funaki NO, Tanaka J, Ohshio G, Onodera H, Maetani S and Imamura M (1998) Cytokeratin 20 mRNA in peripheral venous blood of colorectal carcinoma patients. *Br J Cancer* **77**: 1327–1332

Futamura M, Takagi Y, Koumura H, Kida H, Tanemura H, Shimokawa K and Saji S (1998) Spread of colorectal cancer micrometastases in regional lymph nodes by reverse transcriptase-polymerase chain reactions for carcinoembryonic antigen and cytokeratin 20. *J Surg Oncol* **68**: 34–40

Gerhard M, Juhl H, Kalthoff H, Schreiber HW, Wagener C and Neumaier M (1994) Specific detection of carcinoembryonic antigen-expressing tumor cells in bone marrow aspirates by polymerase chain reaction. *J Clin Oncol* **12**: 725–729

Ghossein RA, Coit D, Brennan M, Zhang ZF, Wang Y, Bhattacharya S, Houghton A and Rosai J (1998) Prognostic significance of peripheral blood and bone marrow tyrosinase messenger RNA in malignant melanoma. *Clin Cancer Res* **4**: 419–428

Gomella LG, Raj GV and Moreno JG (1997) Reverse transcriptase polymerase chain reaction for prostate specific antigen in the management of prostate cancer [see comments]. *J Urol* **158**: 326–337

Hiraga H, Nojima T, Abe S, Sawa H, Yamashiro K, Yamawaki S, Kaneda K and Nagashima K (1998) Diagnosis of synovial sarcoma with the reverse transcriptase-polymerase chain reaction: analyses of 84 soft tissue and bone tumors. *Diagn Mol Pathol* **7**: 102–110

Jung R, Ahmad-Nejad P, Wimmer M, Gerhard M, Wagener C and Neumaier M (1997*a*) Quality management and influential factors for the detection of single metastatic cancer cells by reverse transcriptase polymerase chain reaction. *Eur J Clin Chem Clin Biochem* **35**: 3–10

Jung R, Lubcke C, Wagener C and Neumaier M (1997*b*) Reversal of RT-PCR inhibition observed in heparinized clinical specimens. *Biotechniques* **23**: 24, 26, 28

Jung R, Kruger W, Hosch S, Holweg M, Kroger N, Gutensohn K, Wagener C, Neumaier M and Zander AR (1998) Specificity of reverse transcriptase polymerase chain reaction assays designed for the detection of circulating cancer cells is influenced by cytokines in vivo and in vitro. *Br J Cancer* **78**: 1194–1198

Kruger W, Krzizanowski C, Holweg M, Stockschlader M, Kroger N, Jung R, Mross K, Jonat W and Zander AR (1996) Reverse transcriptase/polymerase chain reaction detection of cytokeratin-19 mRNA in bone marrow and blood of breast cancer patients. *J Cancer Res Clin Oncol* **122**: 679–686

Moll R, Franke WW, Schiller DL, Geiger B and Krepler R (1982) The catalog of human cytokeratins: patterns of expression in normal epithelia, tumors and cultured cells. *Cell* **31**: 11–24

Moll R, Zimbelmann R, Goldschmidt MD, Keith M, Laufer J, Kasper M, Koch PJ and Franke WW (1993) The human gene encoding cytokeratin 20 and its expression during fetal development and in gastrointestinal carcinomas. *Differentiation* **53**: 75–93

Neumaier M, Gerhard M and Wagener C (1995) Diagnosis of micrometastases by the amplification of tissue-specific genes. *Gene* **159**: 43–47

Pantel K, Izbicki J, Passlick B, Angstwurm M, Haussinger K, Thetter O and Riethmuller G (1996) Frequency and prognostic significance of isolated tumour cells in bone marrow of patients with non-small-cell lung cancer without overt metastases. *Lancet* **347**: 649–653

Soeth E, Roder C, Juhl H, Kruger U, Kremer B and Kalthoff H (1996) The detection of disseminated tumor cells in bone marrow from colorectal-cancer patients by a cytokeratin-20-specific nested reverse-transcriptase-polymerase-chain reaction is related to the stage of disease. *Int J Cancer* **69**: 278–282

Soeth E, Vogel I, Roder C, Juhl H, Marxsen J, Kruger U, Henne-Bruns D, Kremer B and Kalthoff H (1997) Comparative analysis of bone marrow and venous blood isolates from gastrointestinal cancer patients for the detection of disseminated tumor cells using reverse transcription PCR. *Cancer Res* **57**: 3106–3110

Traweek ST, Liu J and Battifora H (1993) Keratin gene expression in non-epithelial tissues. Detection with polymerase chain reaction. *Am J Pathol* **142**: 1111–1118

Tschentscher P, Wagener C and Neumaier M (1997) Sensitive and specific cytokeratin 18 reverse transcription-polymerase chain reaction that excludes amplification of processed pseudogenes from contaminating genomic DNA. *Clin Chem* **43**: 2244–2250

Weitz J, Kienle P, Lacroix J, Willeke F, Benner A, Lehnert T, Herfarth C and von Knebel Doeberitz M (1998) Dissemination of tumor cells in patients undergoing surgery for colorectal cancer. *Clin Cancer Res* **4**: 343–348

Wyld DK, Selby P, Perren TJ, Jonas SK, Allen-Mersh TG, Wheeldon J and Burchill SA (1998) Detection of colorectal cancer cells in peripheral blood by reverse-transcriptase polymerase chain reaction for cytokeratin 20. *Int J Cancer* **79**: 288–293

*© 1999 Cancer Research Campaign*
